# Skin autofluorescence is associated with rapid renal function decline in subjects at increased risk of coronary artery disease

**DOI:** 10.1371/journal.pone.0217203

**Published:** 2019-05-22

**Authors:** Chun-Cheng Wang, Ming-Yi Shen, Kuan-Cheng Chang, Guei-Jane Wang, Shu-Hui Liu, Chiz-Tzung Chang

**Affiliations:** 1 Graduate Institute of Clinical Medical Science, China Medical University, Taichung, Taiwan; 2 Division of Cardiovascular Medicine, Department of Internal Medicine, China Medical University Hospital, Taichung, Taiwan; 3 Cardiovascular Research Laboratory, China Medical University Hospital, Taichung, Taiwan; 4 College of Medicine, China Medical University, Taichung, Taiwan; 5 Division of Nephrology, Department of Internal Medicine, China Medical University Hospital, Taichung, Taiwan; Shanghai Institute of Hypertension, CHINA

## Abstract

Skin autofluorescence (AF) has been validated as a tool for estimating tissue advanced glycation end products (AGEs) accumulation and predicting long-term cardiovascular outcomes. However, whether measurements of skin AF could predict renal function decline remains controversial. From April, 2014 to April, 2015, we enrolled 245 subjects with at least two conventional risk factors for coronary artery disease (CAD). All were measured for body height and weight, blood pressure, plasma creatinine level, and skin AF at the start of the study. Baseline demographics and laboratory tests data were obtained by chart reviews and patient interviews. Serial plasma creatinine levels were followed regularly every 6–12 months for 2 years. In a stepwise multivariate linear regression analysis, skin AF, was an independent factor for predicting the relative renal function decline rate after adjustment of multiple covariates (ß = -0.036±0.016; *p* = 0.03). Subgroups analysis revealed that skin AF was a significant factor for relative renal function decline rate in subgroups of age < 65 years (ß = -0.068±0.024; *p* = 0.02), male sex (ß = -0.053±0.016; *p*< 0.01), body mass index≧25 Kg/m^2^(ß = -0.042±0.021; *p* = 0.04), and estimated glomerular filtration rate ≥ 60 ml/min/1.73m^2^(ß = -0.043±0.020; *p* = 0.04). However, only an interaction between skin AF and age attained significance (*p* for interaction = 0.04). Skin AF is a useful predictor for renal function decline in patients at increased risk of CAD.

## Introduction

Advanced glycation end products (AGEs) is formed by nonenzymatic binding between a reducing suger and an amine group from a protein, lipid, or amino acid, which is called the “Maillard reaction”[1]. AGEs formation requires hyperglycemia and increased oxidative stress. Enhanced AGE formation may accumulate in the skin, cardiac, renal, vascular, and neurological systems. Thus, the pathological significance of AGEs accumulation could be systemic[1]. In the cardiovascular system, the AGEs can crosslink with collagen, and elastin, leading to arterial stiffness and impaired myocardial relaxation[2,3]. They can also interact with a receptor for AGEs (RAGE), leading to increased production of reactive oxidative species, suppression of endothelial nitric oxide synthase, reduced production of nitric oxide, and endothelial dysfunction[4]. In the kidney, activation of RAGE with AGEs or other oxidized, dityrosine-containing protein products may induce podocyte apoptosis, generation of monocyte chemoattractant peptide-1, and transforming growth factor-ß, leading to albuminuria, and glomerular sclerosis[5,6]. Treatment with an AGEs breaker reportedly reduced renal glomerulosclerosis in streptozocin-induced diabetic rats[7]. Therefore, AGEs may play a major role in renal toxicity.

Skin autofluorescence (AF) is a well-validated method of estimating skin AGEs accumulation[8]. It’s application in the prediction of mortality, adverse cardiovascular outcomes, and microalbuminuria has been validated[9–11].

Whether skin autofluorescence could be used as a marker for rapid renal function decline in patients at increased risk of coronary artery disease (CAD) has been less discussed. To address this issue, we conducted a prospective cohort study with post hoc analysis to evaluate the association between skin AF values and renal function decline rate in a 2-year follow-up period.

## Material and method

### Study design

A total of 245 patients who have at least 2 conventional risk factors for CAD were recruited from out-patient department from April, 2014 to April, 2015. The conventional risk factors for CAD included presence of chronic hypertension (HTN), hyperlipidemia, diabetes mellitus (DM), smoking, male ≧45 years old, female ≧50 years old, and a family history of premature CAD. DM was defined as a plasma level of glycohemoglobin ≥6.5% or the use of hypoglycemic medications for over 6 months. HTN was defined as a series of at least three systolic blood pressure (SBP) measurements ≥ 140 mmHg or diastolic blood pressure (DBP) measurements ≥90 mmHg at office or the use of anti-hypertensive medications for over 6 months. Hyperlipidemia was defined as a plasma level of total cholesterol >200 mg/dL, low-density lipoprotein cholesterol >130 mg/dL, triglyceride >150 mg/dL, or the use of lipid-lowering medications for over 6 months.[12] We excluded patients with past histories of ischemic heart disease, peripheral arterial occlusive disease (PAOD), cerebrovascular disease, left ventricular systolic dysfunction, moderate to severe valvular heart disease, cardiomyopathy, pericardial disease, and any significant tachy- or brady-arrhythmia. Patients with terminal cancer, psychiatric diseases, unconsciousness, active infection, bed-ridden status, liver cirrhosis, uremia under renal replacement therapy were also excluded from the study. All patients were measured for body height and weight, blood pressure, and skin AF at the start of the study. Each patient had regular follow-up visits every 3 months for up to 2 years in the outpatient department. Information on baseline comorbidities, laboratory data, and medications were obtained from chart reviews and patient interviews. The study protocol has been reviewed and approved by the ethical committee of Taichung Tzu-Chi Buddhist General Hospital (REC103-06). All patients signed informed consent before the enrollment of the study. The study protocol involving human subjects is in accordance with the 1964 Helsinki declaration and its amendament.

## Blood pressure measurements

Patients rested for at least 10 minutes before office measurements and were then instructed to be in a sitting position. Blood pressure measurement was performed with a mercury sphygmomanometer, and a cuff (size 12.5x40cm) was inflated to above the systolic blood pressure (SBP) to occlude the brachial artery. A stethoscope was held just below the cuff for auscultating the palpable pulse. As the cuff is gradually deflated, pulse blood flow is reestablished. The appearance of a clear tapping sound corresponding to the appearance of a palpable pulse (Korotkoff’s sound phase I). The onset of phase I Korotkoff’s sound corresponds to the systolic blood pressure. The disappearance of the Korotkoff’s sound (phase V) corresponds to the diastolic blood pressure (DBP)[13].

### Renal function measurements

All patients received repeat serum creatine measurements at the start of the study and every 6–12 months for 2 years. Serum creatine levels were determined using an enzymatic assay. The estimated glomerular filtration rate (eGFR) was calculated according to the Modification of Diet in Renal Disease (MDRD) Study equation[14]. To calculate the rate of absolute renal function decline, we subtracted the last serum creatine level from the serum creatine level at baseline and then divided it by the time interval between the two visits[15]. To consider the influence of baseline renal function, we defined the rate of relative renal function decline as absolute renal function decline rate/baseline renal function. Rapid renal function decline rate was defined as an absolute eGFR decline rate of > 3 ml/min/1.73m^2^ per year[16].

### Skin AF measurement

The protocol of skin AF measurements has been mentioned in detail elsewhere[17–18]. In brief, the amount of AGEs accumulation in the skin was estimated with an AGE reader (DiagnOptics, Groningen, the Netherlands). Skin AF was measured on the volar side of the forearm, approximately 10 cm below the elbow. The AGEs reader illuminates at a skin surface of 4 cm^2^ with ultraviolet light wavelengths between 300-420nm (peak intensity 370nm). The emitted and reflected light intensity were measured with a spectrometer in the range of 300-600nm. Skin AF (expressed in arbitrary units, AU) was calculated as the average intensity of the emitted light (wavelengths between 420nm to 600nm) divided by the average intensity of reflected light (wavelengths between 300-420nm), multiplied by 100.

### Statistical analysis

We divided the study participants into two groups according to the absolute renal function decline rate. Differences in baseline demographics between the two groups were determined using theχ^2^ test or the Student’s t-test, as appropriate. Univariate linear regression analysis and logistic regression analysis were performed to determine significant factors associated with rate of rapid renal function decline and rate of relative renal function decline. Factors with *p*< 0.05 in the univariate analysis, age, sex, risk factors potentially influencing renal function such as hypertension, diabetes, hyperlipidemia, body mass index (BMI), glycohemoglobin, medications such as statins, non-steroidal anti-inflammatory drugs (NSAIDs), and angiotensinogen converting enzyme inhibitors/Angiotensin II type 1 receptor blocker (ACEI/ARBs) were identified for application in a multivariate linear regression analysis model and logistic regression analysis model. Considering the potential interaction between skin AF and age, sex, diabetes, BMI and renal function at baseline, we further analyzed whether skin AF remained an independent factor in association with renal function decline in different subgroups. One-way analysis of variance (ANOVA) with post-hoc analysis with Bonferroni method was applied to compare differences of renal function decline across skin AF tertiles. All statistical analyses were performed using the IBM Statistical Package for Social Science (SPSS, version 16.0). A *p*-value < 0.05 was considered as statistical significance.

## Results

The baseline demographics of the whole study population and comparisons between the rapid renal function decline rate group (n = 83), and the non-rapid renal function decline rate group (n = 162) are presented in Table 1. The characteristics of the whole study population included 64.48±12.24 years, more male sex (60.41%), and baseline eGFR 66.70±18.58 (mL/min/1.73m^2^), indicating earlier stages of chronic kidney disease. Compared with those in the non-rapid renal function decline rate group, patients in the rapid renal function decline rate group were significantly older (68.89 ± 11.26 years vs. 62.22 ± 12.14 years; *p* < 0.01), comprised a higher proportion of women (49.40% vs. 34.57%; *p* = 0.02), had higher skin AF values (2.46 ± 0.38 AU vs. 2.25 ± 0.38 AU; *p* < 0.01), and lower BMI levels (25.44 ± 4.04 kg/m2 vs. 26.52 ± 3.90 kg/m2; *p* = 0.04).

**Table 1:** Comparisons of baseline demographics between the rapid renal function decline rate and the non-rapid renal function decline rate groups.

	Overall (n = 245)	Non-rapid renal function decline rate (n = 162)	Rapid renal function decline Rate (n = 83)	*p* value
Age (years)	64.48±12.24	62.22±12.14	68.89±11.26	<0.01
Sex (M/F)	148/97	106/56	42/41	0.02
SBP (mmHg)	138.71±16.75	138.71±17.00	138.71±16.36	0.997
DBP (mmHg)	83.42±11.11	84.44±11.09	81.43±10.95	0.045
PP (mmHg)	55.29±12.33	54.27±12.17	57.27±12.45	0.07
Mean BP (mmHg)	101.85±11.92	102.53±12.06	100.52±11.61	0.21
BMI (kg/m^2^)	26.16±3.98	26.52±3.90	25.44±4.04	0.04
Skin AF (AU)	2.32±0.39	2.25±0.38	2.46±0.38	<0.01
Hypertension	196(80%)	130 (80.2%)	66 (79.5%)	0.89
DM				0.70
None	158(64.49%)	106 (65.4%)	52 (62.7%)	
OHA	76(31.02%)	50 (30.9%)	26 (31.3%)	
Insulin	11(4.49%)	6 (3.7%)	5 (6.0%)	
Hyperlipidemia	179(73.06%)	115 (71.0%)	64 (77.1%)	0.31
Smoking				0.89
None	173(70.61%)	116 (71.6%)	57 (68.7%)	
Ever	44(17.96%)	28 (17.3%)	16 (19.3%)	
Active	28(11.43%)	18 (11.1%)	10 (12.0%)	
Medications:				
Antiplatelets	160(65.31%)	97 (59.9%)	63 (75.9%)	0.01
CCBs	103(42.04%)	68 (42.0%)	35 (42.2%)	0.98
ß-blockers	101(41.22%)	65 (40.1%)	36 (43.4%)	0.63
ACEI/ARBs	150(61.22%)	104 (64.2%)	46 (55.4%)	0.18
Statins	131(94.29%)	80 (49.4%)	51 (61.4%)	0.07
NSAIDs				0.02
None	196(80%)	134 (82.7%)	62 (74.7%)	
≤3 months	18(7.35%)	14 (8.6%)	4 (4.8%)	
>3 months	31(12.65%)	14 (8.6%)	17 (20.5%)	
GFR (mL/min/1.73m^2^)	66.70±18.58	68.61±18.64	62.95±17.96	0.02
Glycohemoglobin (%)	6.31±1.04	6.28±1.00	6.35±1.12	0.60
Relative renal function decline rate (%/year)	-2.34%±9.70%	2.90%±5.78%	-12.59%±7.34%	<0.01

M: male; F: female; SBP: systolic blood pressure; DBP: diastolic blood pressure; PP: pulse pressure; Skin AF: skin autofluorescence; AU: arbitrary unit; BMI: Body mass index; DM: diabetes mellitus; OHAs: oral hypoglycemic agents; HBA1C: glycohemoglobin; CCBs: calcium channel blockers; ACEI: angiotensinogen converting enzyme inhibitors; ARBs: Angiotensin II type 1 receptor blocker; NSAIDs: Nonsteroidal anti-inflammatory drugs; GFR: glomerular filtration rate.

To determine whether skin AF could be an independent factor for rapid renal function decline rate, we first identified factors significantly associated with rapid renal function decline rate in the univariate analysis, namely age, sex, skin AF, BMI, DBP, eGFR, antiplatelets, and NSAIDs. All factors were then entered into a multivariate logistic regression model. After adjustment for age, sex, DBP, pulse pressure (PP), BMI, DM, glycohemoglobin, Hypertension, hyperlipidemia, antiplatelets, ACEIs/ARBs, statins, and NSAIDs, skin AF and NSAIDs remained independent factors for predicting rapid renal function decline (adjusted odds ratio [OR]: 3.22, 95% confidence interval [CI]: 1.40–7.40, *p* < 0.01; adjusted OR: 1.57, 95% CI: 1.02–2.42, *p* = 0.04) (Table 2).

**Table 2:** Univariate and multivariate logistic regression analysis to identify independent factors associated with rapid renal function decline.

	Crude O.R.	95% C.I.	P value	Adjusted O.R.	95% C.I.	P value
Age (year)	1.05	1.02–1.08	< 0.01	1.02	0.99–1.06	0.21
Sex (Female vs. Male)	1.85	1.08–3.17	0.03	1.57	0.84–2.92	0.15
Skin AF (A.U.)	4.42	2.09–9.35	<0.01	3.22	1.40–7.40	<0.01
SBP (mmHg)	1.00	0.98–1.02	0.997	-	-	-
DBP (mmHg)	0.98	0.95–1.00	0.047	1.00	0.97–1.02	0.75
Mean BP (mmHg)	0.99	0.96–1.01	0.21	-	-	-
PP (mmHg)	1.02	1.00–1.04	0.07	1.01	0.99–1.04	0.36
BMI (kg/m2)	0.93	0.86–0.999	0.046	0.95	0.87–1.04	0.26
GFR (mL/min/1.73m^2^)	0.98	0.97–0.996	0.01	0.99	0.98–1.01	0.41
DM	1.13	0.65–1.96	0.67	0.87	0.39–1.92	0.73
Glycohemoglobin	1.07	0.83–1.37	0.60	1.02	0.72–1.44	0.93
Hypertension	0.96	0.50–1.85	0.89	-	-	-
Hypelipidemia	1.38	0.75–2.55	0.31	1.79	0.72–4.47	0.21
Smoking	1.09	0.74–1.59	0.68	-	-	-
Antiplatelets	2.11	1.17–3.82	0.01	1.61	0.80–3.23	0.18
ß-blockers	1.14	0.67–1.95	0.63	-	-	-
CCBs	1.01	0.59–1.72	0.98	-	-	-
ACEIs/ARBs	0.69	0.40–1.19	0.18	0.77	0.41–1.46	0.43
Statins	1.63	0.95–2.80	0.07	1.38	0.64–2.96	0.41
NSAIDs	1.49	1.03–2.16	0.04	1.57	1.02–2.42	0.04

O.R.: Odds ratio; C.I.: confidence interval; Skin AF: skin autofluorescence; A.U.: arbitrary unit; SBP: systolic blood pressure; DBP: diastolic blood pressure; PP: pulse pressure; BMI: Body mass index; GFR: glomerular filtration rate; DM: diabetes mellitus; CCBs: calcium channel blockers; ACEI: angiotensinogen converting enzyme inhibitors; ARBs: Angiotensin II type 1 receptor blocker; NSAIDs: Nonsteroidal anti-inflammatory drugs. DM, smoking, and NSAIDs were regarded as rank variable.

Using the relative renal function decline rate as a dependent variable, we observed that age, skin AF, DBP, PP, BMI, eGFR, and antiplatelet treatment were significant factors associated with the renal function decline rate. Multivariate linear regression analysis revealed that skin AF remained significantly associated with the renal function decline rate after adjustment for age, sex, DBP, PP, eGFR, DM, glycohemoglobin, Hyperlipidemia, antiplatelet, ACEIs/ARBs, statins, NSAIDs treatment. An increase of 1 AU of skin AF explained a decrease of 3.6%/year in the relative renal function decline rate (*p* = 0.03) (Table 3).

**Table 3:** Univariate and multivariate linear regression analysis to identify independent factors associated with rate of relative renal function decline.

	Unstandardized coefficient B	S.E.	P value	Unstandardized coefficient B	S.E.	P value
Age (year)	-0.002	0.000	< 0.01	-0.001	0.001	0.09
Sex (F vs, M)	-0.021	0.013	0.09	-0.008	0.013	0.53
Skin AF (A.U.)	-0.06	0.015	< 0.01	-0.036	0.016	0.03
SBP (mmHg)	0.000	0.000	0.97	-	-	-
DBP (mmHg)	0.001	0.001	<0.01	0.001	0.001	0.30
PP (mmHg)	-0.001	0.000	0.02	-0.001	0.001	0.32
BMI (kg/m2)	0.004	0.002	0.02	0.002	0.002	0.18
eGFR (mL/min/1.73m^2^)	0.001	0.000	<0.01	0.001	0.000	0.05
DM	-0.011	0.013	0.40	0.008	0.016	0.60
Glycohemoglobin (%)	-0.01	0.006	0.10	-0.01	0.007	0.16
Hypertension	0.009	0.016	0.56	-	-	-
Hypelipidemia	-0.02	0.014	0.16	-0.045	0.017	<0.01
Smoking	-0.007	0.009	0.44	-	-	-
Antiplatelets	-0.025	0.013	0.05	-0.005	0.014	0.71
ß-blockers	-0.014	0.013	0.27	-	-	-
CCBs	0.005	0.013	0.67	-	-	-
ACEIs/ARBs	0.019	0.013	0.14	0.019	0.013	0.15
Statins	-0.005	0.012	0.66	0.022	0.015	0.15
NSAIDs	-0.012	0.009	0.20	-0.011	0.009	0.21

S.E.: Standard error; F: female; M: male; Skin AF: skin autofluorescence; A.U.: arbitrary unit; SBP: systolic blood pressure; DBP: diastolic blood pressure; PP: pulse pressure; BMI: Body mass index; eGFR: estimated glomerular filtration rate; DM: diabetes mellitus; CCBs: calcium channel blockers; ACEI: angiotensinogen converting enzyme inhibitors; ARBs: Angiotensin II type 1 receptor blocker; NSAIDs: Nonsteroidal anti-inflammatory drugs. DM, smoking, and NSAIDs were regarded as rank variable.

**Fig 1:**
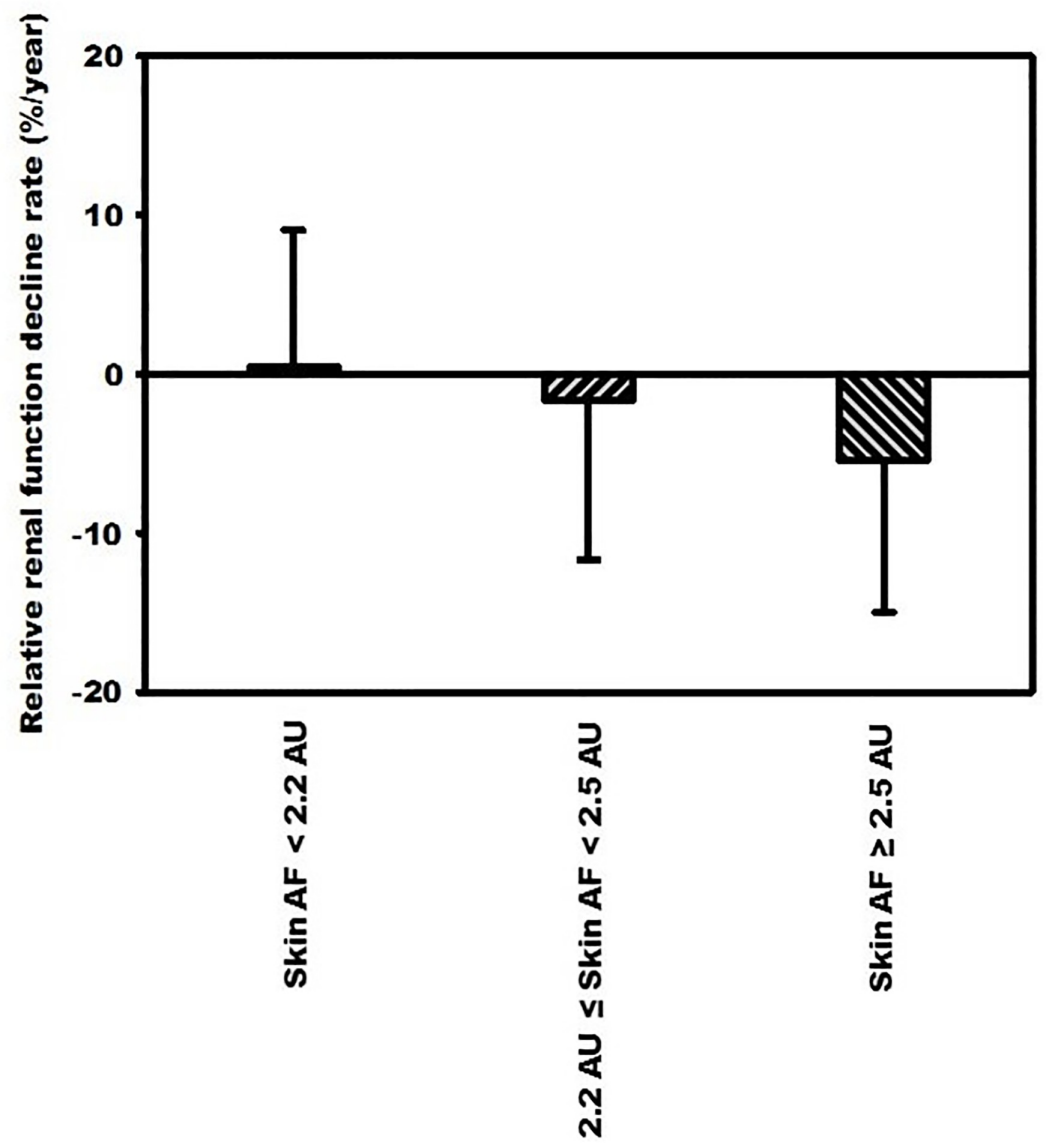
Skin autofluorescence levels were divided into tertiles and the relative renal function decline rate progressively increased across all tertiles.

We further divided skin AF values into tertiles. The rate of relative renal function decline increased progressively across all tertiles (skin AF < 2.2 AU vs. 2.2 AU ≤ skin AF < 2.5 AU vs.; 0.43% ± 8.61% /year vs. -1.65% ± 10.02%/year vs. -5.38% ± 9.60%/year; *p* < 0.01). Post hoc analysis with multiple comparisons revealed that the third tertile of skin AF group had significantly higher relative renal function decline rate than the first tertile (-5.38% ± 9.60%/year vs. 0.43% ± 8.61% /year; *p* < 0.01) (Fig 1).

We further examined factors associated with skin AF values in our cohort. Age, PP, BMI, eGFR, DM, glycohemoglobin, and smoking are factors significantly associated with skin AF values in the univariate linear regression model. When age, PP, BMI, eGFR, DM, and smoking were entered into the multivariate linear regression model, only age, DM, and eGFR were independent factors associated with skin AF value (Table 4).

**Table 4:** Univariate and multivariate linear regression analysis to identify independent factors associated with skin autofluorescence.

	Unstandardized coefficient B	S.E.	P value	Unstandardized coefficient B	S.E.	P value
Age (year)	0.011	0.002	< 0.01	0.008	0.002	<0.01
Sex (F vs, M)	-0.004	0.052	0.94	-	-	-
SBP (mmHg)	0.001	0.002	0.49	-	-	-
DBP (mmHg)	-0.003	0.002	0.13	-	-	-
Mean BP (mmHg)	-0.001	0.002	0.54	-	-	-
PP (mmHg)	0.005	0.002	0.02	0.000	0.002	0.81
BMI (kg/m^2^)	-0.017	0.006	< 0.01	-0.012	0.006	0.06
eGFR (mL/min/1.73m^2^)	-0.004	0.001	<0.01	-0.003	0.001	0.03
DM	0.144	0.052	<0.01	0.126	0.050	0.01
Glycohemoglobin (%)	0.058	0.024	0.02	-	-	-
Hypertension	0.021	0.063	0.74	-	-	-
Hypelipidemia	-0.007	0.057	0.90	-	-	-
Smoking	0.098	0.036	<0.01	0.069	0.035	0.05
Antiplatelets	0.05	0.053	0.34	-	-	-
ß-blockers	0.037	0.051	0.47	-	-	-
CCBs	0.041	0.051	0.42	-	-	-
ACEIs/ARBs	-0.005	0.052	0.92	-	-	-
Statins	0.091	0.050	0.07	-	-	-
NSAIDs	0.000	0.037	1.00	-	-	-

S.E.: Standard error; F: female; M: male; SBP: systolic blood pressure; DBP: diastolic blood pressure; PP: pulse pressure; BMI: Body mass index; eGFR: estimated glomerular filtration rate; DM: diabetes mellitus; HBA1C: Glycohemoglobin; CCBs: calcium channel blockers; ACEI: angiotensinogen converting enzyme inhibitors; ARBs: Angiotensin II type 1 receptor blocker; NSAIDs: Nonsteroidal anti-inflammatory drugs.

Considering the potential interactions between skin AF and age, sex, DM, renal function at baseline and BMI, we performed subgroup analysis to determine whether skin AF remained an independent factor for renal function decline in different clinical settings. The result is presented in Table 5. After adjustment for age, DBP, PP, BMI, eGFR, DM, Hypertension, hyperlipidemia, and antiplatelets, ACEIs/ARBs, NSAIDs, statins treatment, skin AF remained significantly associated with the renal function decline rate among subgroups of age < 65 years, male sex, BMI ≥ 25 Kg/m^2^, and eGFR ≥ 60 ml/min/1.73m^2^. However, only the interaction between skin AF and age attained significance (*p* = 0.04). There was no significant interaction among skin AF and sex (*p* = 0.6), DM (*p* = 0.5), eGFR (*p* = 0.41), BMI (*p* = 0.87).

**Table 5:** Subgroups analyses to investigate skin autofluorescence as an independent factor for rate of renal function decline.

		Unstandardized coefficient B	S.E.	*p* value	*p* for interaction
Age (years)	Age < 65 (n = 129)	-0.068	0.024	<0.01	0.04
	Age≧65 (n = 118)	-0.019	0.030	0.53	
Sex	Male (n = 148)	-0.053	0.016	< 0.01	0.60
	Female (n = 97)	-0.025	0.034	0.47	
DM	with (n = 87)	-0.056	0.029	0.057	0.50
	without (n = 158)	-0.028	0.021	0.18	
eGFR (mL/min/1.73m^2^)	eGFR≧60 (n = 149)	-0.043	0.020	0.04	0.41
	eGFR < 60 (n = 96)	-0.039	0.028	0.18	
BMI (Kg/m2)	BMI < 25 (n = 102)	-0.025	0.029	0.39	0.87
	BMI≧25 (n = 143)	-0.042	0.021	0.04	

S.E.: standard error; DM: diabetes mellitus; eGFR: estimated glomerular filtration rate, BMI: Body mass index.

## Discussion

### Proposed mechanisms of skin AF as a tool for predicting renal function decline

In our study, we demonstrated that skin AF could predict renal function decline in a 2-year follow-up period in patients at increased risk of CAD. The possible explanations for this are as follows: First, AGEs accumulation within the glomeruli could induce activation of RAGE and apoptosis of podocytes[5], and upregulation of genes coding for extracellular matrix such as type IV collagen, laminin, and transforming growth factor-ß1 (TGF- ß1) in the glomeruli[19]. Treatment with an AGEs breaker was shown to reduce renal fibrosis in streptozocin-induced diabetic rats[20]. Second, our study demonstrated that PP is associated with skin AF (Table 4), supporting the general notion that AGEs accumulation within vessels could lead to increased arterial stiffness[3]. Increased PP, as a marker for arterial stiffness, has been validated as a predictor of renal function decline[15,21,22]. The glomerular capillary is positioned between afferent and efferent arterioles. Under physiological condition, the resistance of the efferent arteriole is higher than that of the afferent arteriole in order to maintain glomerular hydrostatic pressure and GFR. Because the resistance of the renal vasculature is low, the pressure drop across afferent arteriole is small. The increased PP could lead to accentuated circumferential and shear wall stress and would be transmitted to the glomeruli, resulting in glomerular barotrauma. In addition, the myogenic tone of the afferent arteriole, which could regulate and counteract the increased PP, is impaired by arterial stiffness[23–25]. Thus, tissue AGEs accumulation may worsen renal function through arterial stiffness. Third, as our published data indicated, skin AF could be used as a marker for inappropriate left ventricular mass and diastolic dysfunction[18]. Diastolic heart failure (HFpEF) has been proposed as a cause of renal function deterioration. The main mechanisms include increased intra-abdominal pressure, increased central venous pressure, activation of renal-angiotensin-aldosterone system (RAA), and sympathetic tone hyperactivity[26].

In addition, compared with subjects with appropriate left ventricular mass, those with inappropriate left ventricular mass had lower cardiac output and reduced ventricular wall mechanics[27]. AGEs may cause renal function deterioration through their accumulation in the myocardium, resulting in abnormal myocardial growth and stiffness.

### Comparisons of our data with previous studies

Previous studies have investigated whether skin AF could be a useful tool for predicting renal function decline and they have reported controversial results[16,28]. This may be explained by differences in study populations, racial differences, and different endpoints of the study designs. Compared with previous studies, our study population was in the subclinical atherosclerosis and earlier CKD stages. Because subjects in more advanced atherosclerosis may have more complicated comorbidities, the importance of AGEs accumulation in relation to renal function deterioration may become less significant. Our study proposed that application of skin AF could be a useful marker for relative renal function decline rate in patients at increased risk of CAD, especially in subjects at age < 65 years, male sex, BMI ≥ 25 Kg/m^2^, and early CKD stage (CKD stage I or II).

### Subgroup analysis to investigate the relation between skin autofluorescence and renal function decline

Subgroup analysis showed skin AF to be an independent factor for relative renal function decline rate in the settings of age < 65 years, male sex, BMI ≧ 25 Kg/m^2^, and eGFR≧60 mL/min/1.73m^2^. Our findings that skin AF was not an independent factor for the subgroups of age≧65 years, female sex, BMI < 25 Kg/m^2^ and baseline eGFR < 60 mL/min/1.73m^2^ require further validation. Due to the limited sample size, our study does not have adequate power to conclude these findings. Nontheless, the results of subgroup analysis further strengthen our hypothesis that skin AF is an independent and useful tool for predicting renal function decline and this result is not confounded by other potential risk factors such as old age, and renal dysfunction.

### Study limitation

Our study has several limitations. First, because this is an observational study, we could only provide an evidence that skin AF is a useful marker for predicting renal function decline. Our study could not establish a cause-effect relationship. Second, we only enrolled participants at increased risk for CAD, and more than 90% of the study subjects belonged to CKD stage I-III. Our study subjects represent a population at subclinical atherosclerosis and early stage of CKD. The result could not be extrapolated to other subjects. Third, we cannot provide data on microalbuminuria, and inflammatory markers, both of which have been validated as independent factors associated with renal function decline[29,30]. Fourth, the correlation between skin AF level and the amount of plasma AGEs concentrations, and skin AGEs accumulation has been validated.[8,31] However, to our knowledge, no study has confirmed that skin AF could be a proxy for renal AGEs accumulation. Fifth, in our study, increased brachial PP failed to be an independent predictor for renal function decline and skin AF value (Tables 3 and 4). It may be explained by the method of measurement of arterial stiffness. We should note that central aortic stiffness, rather than peripheral arterial stiffness, as a more well validated marker and prognosticator for clinical adverse cardiovascular outcome. The golden standard for measurement of central aortic stiffness is carotid-femoral pulse wave velocity (cfPWV). Brachial pulse pressure cannot represent central pulse pressure and pulse pressure cannot represent PWV because PP is more influenced by pulse wave reflection.[32] Sixth, the major limitation of our study is its relatively small sample size and mid-term follow-up period. Our study result should be further verified with a larger sample size and longer follow-up period.

## Conclusion

We proposed that skin AF could be an independent factor for renal function decline in patients at increased risk of CAD.

## Supporting information

S1 Database (XLS)
